# Determination of Strength Exercise Intensities Based on the Load-Power-Velocity Relationship

**DOI:** 10.2478/v10078-011-0020-2

**Published:** 2011-07-04

**Authors:** Daniel Jandačka, Petr Beremlijski

**Affiliations:** 1 Human Motion Diagnostics Center, University of Ostrava, Czech Republic; 2 Department of Applied Mathematics, Faculty of Electrical Engineering and Computer Science, Technical University of Ostrava, Czech Republic

**Keywords:** biomechanics, optimization, bench press, soccer

## Abstract

The velocity of movement and applied load affect the production of mechanical power output and subsequently the extent of the adaptation stimulus in strength exercises. We do not know of any known function describing the relationship of power and velocity and load in the bench press exercise. The objective of the study is to find a function modeling of the relationship of relative velocity, relative load and mechanical power output for the bench press exercise and to determine the intensity zones of the exercise for specifically focused strength training of soccer players. Fifteen highly trained soccer players at the start of a competition period were studied. The subjects of study performed bench presses with the load of 0, 10, 30, 50, 70 and 90% of the predetermined one repetition maximum with maximum possible speed of movement. The mean measured power and velocity for each load (kg) were used to develop a multiple linear regression function which describes the quadratic relationship between the ratio of power (W) to maximum power (W) and the ratios of the load (kg) to one repetition maximum (kg) and the velocity (m•s^−1^) to maximal velocity (m•s^−1^). The quadratic function of two variables that modeled the searched relationship explained 74% of measured values in the acceleration phase and 75% of measured values from the entire extent of the positive power movement in the lift. The optimal load for reaching maximum power output suitable for the dynamics effort strength training was 40% of one repetition maximum, while the optimal mean velocity would be 75% of maximal velocity. Moreover, four zones: maximum power, maximum velocity, velocity-power and strength-power were determined on the basis of the regression function.

## Introduction

In sports where acyclic movement is used to reach the maximal performance, such as throws, jumps, kicks or fast changes in the direction, rapid increase or decrease in speed, it is often necessary to produce maximum mechanical power output. The method of dynamics effort strength training uses manipulation of the movement velocity and applied load to reach the maximum mechanical power output and subsequently to affect the extent of the adaptation stimulus. For example, Kovaleski et al. (1992) concluded that when performing velocity spectrum type training, performing faster speed sets early in the exercise session will produce a greater average power. The intensity of exercise should then be logically set on the basis of the knowledge of relationships between the maximum power, velocity and applied load during strength exercises.

One of the first people who tried to express the relationships of velocity-strength and power (the velocity of releasing heat and performing work) was Nobel Laureate Archibald Hill (1938). However, his studies only focus on isolated muscles of frogs and stimulated contractions. From the practical point of view, we are mainly interested in the relationship of the mechanical power output (we will omit heat), external load and velocity in a complex movement. Hill’s (1938) equation implies that maximum mechanical muscle power output is reached approximately by one third of the maximum strength and velocity. Using this result to optimize the load during the training of athlete or to optimize the sports performance is very difficult. It is necessary to acknowledge that frogs have relatively larger jumping abilities than humans. During sports movements, many muscles with different courses of muscle fibers, tendon properties and proprioreceptor behavior are involved at the same time. There is a difference in the production of the power output between a volitional and stimulated contraction and fatigue plays a significant role in the sports performance.

The production of maximum mechanical power output during a complex human movement is a value which is influenced by several neural and intramuscular factors (Kraemer & Newton 2000). In spite of the large number of factors influencing the maximum power output during a complex volitional human movement, it is important to know the velocity of energy release both during strength exercises and individual sports movements for practical reasons. The description of the velocity-load and mechanical power output relationship may help in rationalization of the determination of optimal stimulus for power or strength training. For example assumption that maximal or near maximal force (very heavy resistance) is required for recruitment of the higher-threshold motor units and optimal strength gains is not supported by the size principle, motor unit activation studies, or resistance training studies (Carpinelli 2008). So far, scientists have mainly focused on the expression of the optimal load to reach maximum mechanical power output (Baker et al. 2001a; Baker et al. 2001b; Cormie et al. 2007; Kawamori et al. 2005; Thomas et al. 2007) description of the relationship of relative load (expressed as percentage of one repetition maximum) and mechanical power output (Jandacka & Vaverka 2008). Jidovtseff et al. (2009) estimated the optimal training zones for strength training on the basis of the knowledge of the load-velocity and load-power relationships. We believe that one functional power-load-velocity relationship would be more convenient for the determination of optimal training zones.

The whole issue of determining the velocity-load-mechanical power output relationship in a complex human movement such as strength exercise gets, however, complicated by contradictions that appear in literature with regard to the method of determining mechanical power output. The methodology used to determine maximal power output has also been suggested to be a contributing factor to discrepancies in the literature (Cormie et al. 2007; Jandacka & Vaverka 2009; Li et al. 2008). Hori et al. (2006) reported four methods which are usually used for determining power output. Methods based on the kinematics of barbell motion assume that the centre of mass of the system mass and barbell move parallel with the lifted barbell. Only the kinetic method assumes that whole parts of the body are moving during the lift. These assumptions logically underestimate or overestimate power output depending on the method used. In this study we used a different approach to determine power output without the assumption of parallel movement of the barbell and centre of mass or the whole body moving during the lift.

As mechanical power output does not only depend on external load (Jandacka & Vaverka 2008), but also on the velocity of the movement during strength exercise, the first aim of this study is to find a function modeling of the relationship of relative velocity, load and mechanical power output. During the optimization we ask what model setting will ensure optimal (maximum or minimum) result under the given conditions (Caldwell 2004). The second objective of our study is to determine the combination of relative velocity and load during the bench press exercise which predetermines maximum mechanical poweroutput in highly trained soccer players. The third objective of the study is to propose exercise intensity zones for resistance training of soccer players on the basis of a created three-dimensional model and optimal combination of velocity and load for maximum mechanical power output.

## Methods

### Subjects

Fifteen highly trained soccer players at the start of a competition period with a mean ± *SD* age, height, and body mass of 26.1 ± 3.9 years, 183.3 ± 6.7 cm, and 78.8 ± 7.2 kg, respectively, were studied. The study protocol was approved by the Ethics and Research Committee of the University of Ostrava. All subjects signed an informed consent form and they were members of the same soccer club.

### Procedures

Each subject visited the laboratory on two separate occasions with a one week rest. In the first session, subjects were given instructions on the techniques of the bench press (Zatsiorsky & Kraemer 2006). The range of motion for each subject was established without chest-touch position and controlled by means of an audible signal at the highest and lowest peak of the motion trajectory. Then, the body height, weight of body and mass of upper extremity segments using the segmental body composition analyzer (TANITA 418 MA, USA) were determined. The first session involved one repetition maximum testing according to the protocol published by Kraemer et al. (2006). The second session involved the measurement of the power output for the bench press while systematically increasing the load 0, 10, 30, 50, 70 and 90% of one repetition maximum (0% of one repetition maximum means the weight of upper extremities). Retro reflective markers were attached to the acromion, greater tubercle of the humerus, medial epicondyle of the humerus, lateral epicondyle of the humerus, styloid process of the radius, styloid process of the ulna, terminal points and the medial point of the barbell. In addition, four light-weight rigid plates holding a triad of markers were attached to the upper arms and forearms. The force plates were set to zero when the subject assumes the initial position on the bench without the load. The athlete assumed the lifting position for the bench press, with the feet placed in the position on the foot holder connected to the bench and a self-selected grip that they had been constantly using in training for this exercise. After collecting a static trail in which they were required to stand in the initial upper position with the barbell, three acceptable trials with each load were collected. An acceptable trial was one in which the subject complied with the range of motion during the lift. The subjects were instructed that the bar must be lowered under control until they reached the bottom position. Upon reaching this position and hearing the audible signal via the FitroDyne Premium device (Fitro, Slovakia), the subjects were required to lift the load with maximal speed. The subjects were not instructed to explode off the bench surface or throw the barbell. A three-minute rest was given between each lift. Three trials with each load were collected. The mean of the three trials was accepted for further analysis.

### Experimental Setup

**Figure 1:**
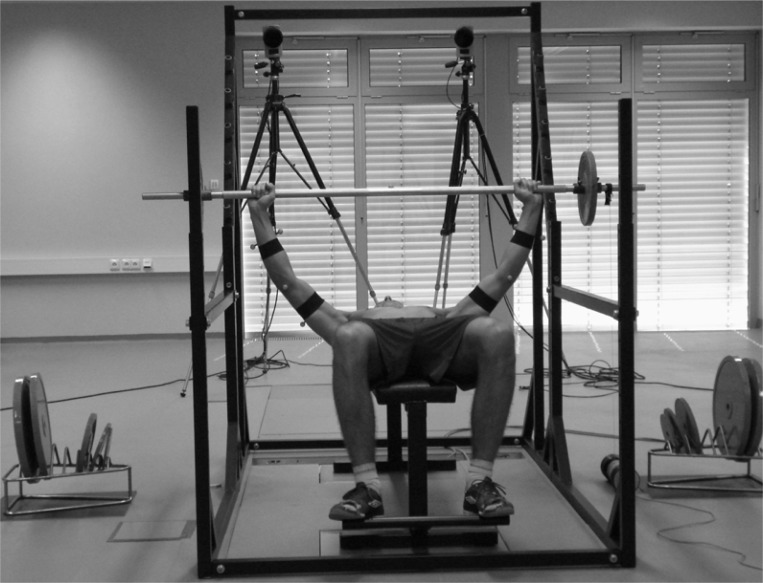
Experimental setup of range of motion, force and velocity measurements during bench press exercises. The bench stands on the force plates. FitroDyne Premium device is connected to the barbell. Infra-red cameras layout and focus on the area of movement. All devices connect to the PC run through an A/D board. Markers are placed on the barbell and upper extremities.

Two force plates (Kistler 9281CA and 9286AA, Switzerland) embedded in the floor and positioned below the bench, sampling at 988 Hz, were used to measure contact forces between the bench and ground during the lift. Three-dimensional upper extremities kinematic data during the bench press were collected at 247 Hz using a seven camera motion capture system (Qualisys Oqus, Sweden). Data from the force plates and the cameras were collected simultaneously. The linear position transducer device (FitroDyne Premium, Slovakia) signaled using a sound that the subject could hear throughout the trial and which changed when the downward movement switched to the upward phase of the movement. Power testing was performed using free weight form techniques (Figure 1).

### Data Analysis

**Figure 2:**
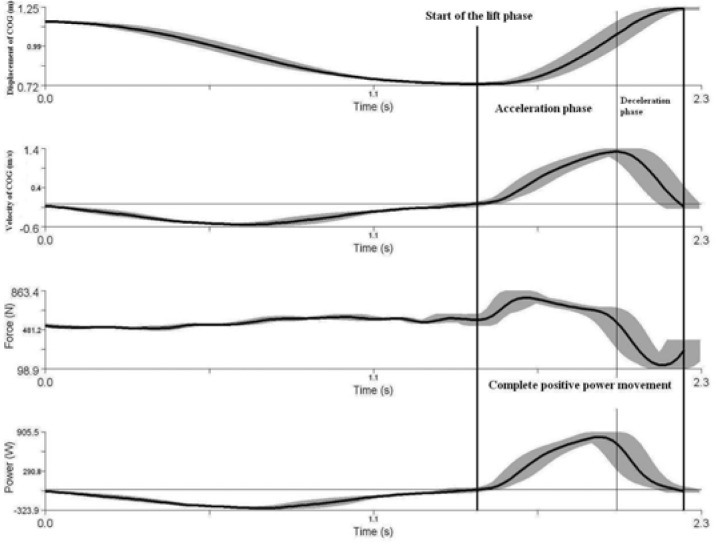
Relationship between centre of gravity displacement (COG of barbell and upper extremities), vertical velocity of COG, vertical ground reaction force, vertical mechanical power and time during bench press with 44-kg load (50% of one repetition maximum). Solid curve represents mean of three trials of one subject and gray area represents standard deviation.

Power (W) was calculated as the product of vertical force (N) and vertical velocity (m•s^−1^) of the center of gravity (COG of system upper extremity segments and barbell). The velocity of the center of gravity (m•s^−1^) was the necessary parameter derived from the visual 3D software. Marker data were processed using Visual 3D software (C-motion, Rockville, MD, USA). All upper extremity segments with the exception of hands were modeled as a frustum of right circular cones whilst the barbell was modeled as a cylinder. The vertical force (N) was obtained as the sum of two vertical ground reaction force (N) signals from two force plates and the weight of the upper extremities (N). The weight of the upper extremities (N) was calculated as a product of mass of the upper extremities (kg) and gravity acceleration (m•s^−2^). The power (W) for each load on each lift was determined. We analyzed the part of the motion which showed positive power output (W). By differentiating the velocity (m•s^−1^) of the center of gravity, the acceleration and deceleration parts of the upward part of the lift were determined (Jandacka & Vaverka 2008) (Figure 2). Thus the mean power (W) for each load (% of one repetition maximum) and lift was determined from the complete positive power movement and from the acceleration phase of the movement as well. Maximum power output (W) was the absolute maximum for all loads. We neglected the horizontal power which was negligibly small for all loads.

### Statistical Analysis

Intra-class correlations for repeated power output measurement was computed from 3 trials with each load. Power output with all loads displayed intraclass correlations coefficients above the minimum acceptable criterion 0.7. The ratio of power (W) to maximum power (W) was determined several times for each subject at each load (kg). The averages of the measured values at each load and velocity were used to develop a multiple linear regression model which describes the quadratic relationship between the ratio of power to maximum power and the ratios of the load and the velocity for two measured data sets. This regression model was made by least squares method implemented in Matlab. The developed model is expressed as

$$\frac{P}{P_{mm}}=b_{1}{\left(\frac{L}{1RM}\right)}^{2}+b_{2}{\left(\frac{v}{v_{mm}}\right)}^{2}+b_{3}\left(\frac{L}{1RM}\right)\cdot \left(\frac{v}{v_{mm}}\right)+b_{4}\left(\frac{L}{1RM}\right)+b_{5}\left(\frac{v}{v_{mm}}\right)+c$$

where *P*_mm_ is mean maximal power (W), *P* is mean power (W), 1RM is maximal load (kg)*, L* is load (kg), *v*_mm_ is mean maximal velocity (m•s^−1^), *v* is mean velocity (m•s^−1^), *b*_1_, *b*_2_, *b*_3_, *b*_4_ and *b*_5_ are the regression coefficients and c is the regression constant. It was verified that the both constructed linear regression models were correct. We used Stagraphics Plus for the verification of key assumptions which are essential to be true. The optimal load and velocity of the regression model were found by the trust region approach combined with the quasi-Newton (Bonnans et al. 2006). We used Levenberg-Marquardt implementation of the trust region approach in Matlab. In the end, we calculated 95% confidence interval by program Statgraphics Plus.

## Results

**Figure 3:**
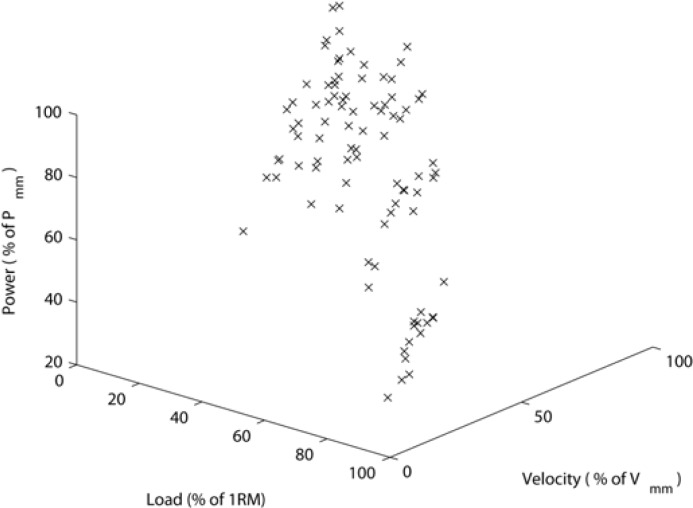
The averages of the mechanical power output calculated from the acceleration phase of the movement. The relative loads and velocities are the explanatory variables. The relative powers are the dependent variables.

The mean ± standard deviation one repetition maximum bench press for a group of fifteen soccer players was 83.3 ± 11.2 kg. The maximal power output *P*_mm_ was 576.0 ± 92.2 W for the acceleration phase of the lift and 448.5 ± 73.7 W for the complete positive power movement. The averages of the mechanical power output calculated from the acceleration phase of the movement are shown in Figure 3.

The linear regression model for these data was expressed as

$$\begin{array}{l} \frac{P}{P_{mm}}=0.02458{\left(\frac{L}{1RM}\right)}^{2}-0.02182{\left(\frac{v}{v_{mm}}\right)}^{2}-0.02695\left(\frac{L}{1RM}\right)\cdot \left(\frac{v}{v_{mm}}\right)+3.998\left(\frac{L}{1RM}\right)+\\ 4.344\left(\frac{v}{v_{mm}}\right)144.3 \end{array}$$

**Figure 4:**
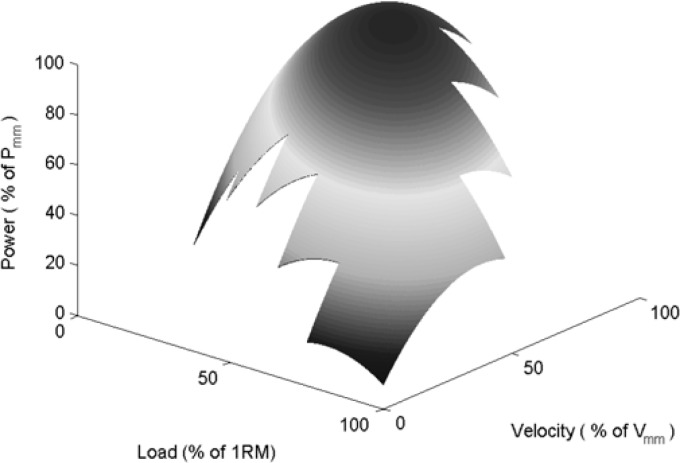
The linear regression model which describes the quadratic relationship between the relative power and the relative load and the relative velocity for the acceleration phase of the movement.

**Figure 5:**
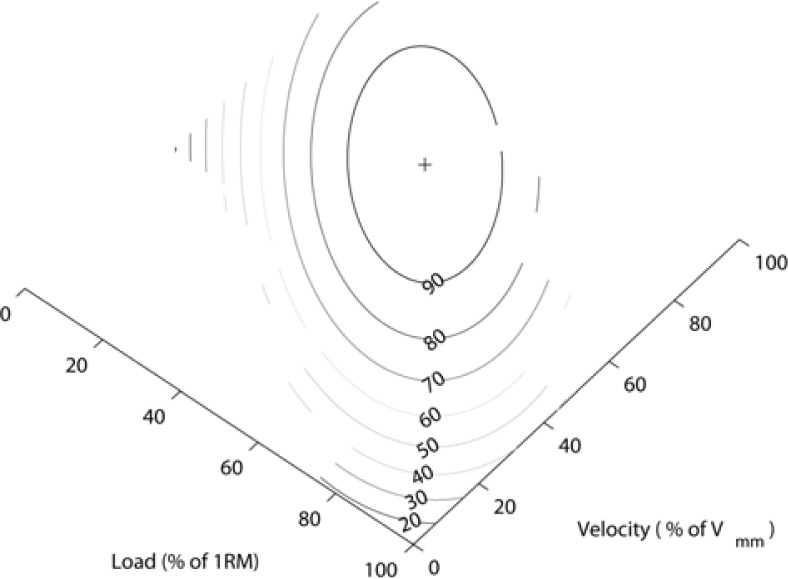
The contour lines of the linear regression model for the acceleration phase of the movement.

Figure 4 presents the developed linear regression model, while Figure 5 presents the contour lines of this model. The optimal load for this model is 40.43% of 1RM, while the optimal velocity is 74.58% of *v*_mm_.

**Figure 6:**
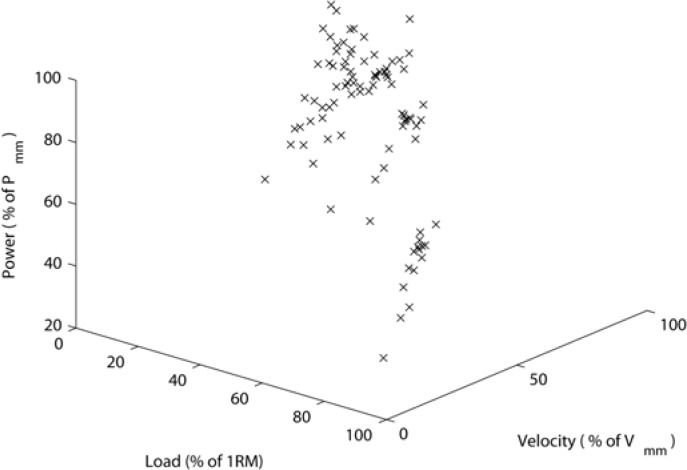
The averages of the mechanical power output calculated from the complete positive power movement. The relative loads and velocities are the explanatory variables. The relative powers are the dependent variables.

The mean values of the mechanical power output calculated from the complete positive power movement are shown in Figure 6.

The linear regression model calculated for the complete positive power movement was expressed as:

$$\begin{array}{l} \frac{P}{P_{mm}}=-0.02626{\left(\frac{L}{1RM}\right)}^{2}-0.0303{\left(\frac{v}{v_{mm}}\right)}^{2}-0.03662\left(\frac{L}{1RM}\right)\cdot \left(\frac{v}{v_{mm}}\right)+5.212\left(\frac{L}{1RM}\right)+\\ 6.094\left(\frac{v}{v_{mm}}\right)-243.9 \end{array}$$

**Figure 7:**
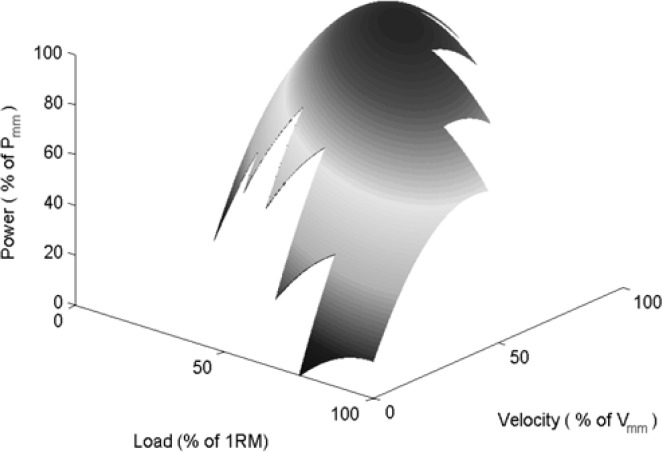
The linear regression model which describes the quadratic relationship between the relative power and the relative load and the relative velocity for the complete positive power movement.

**Figure 8:**
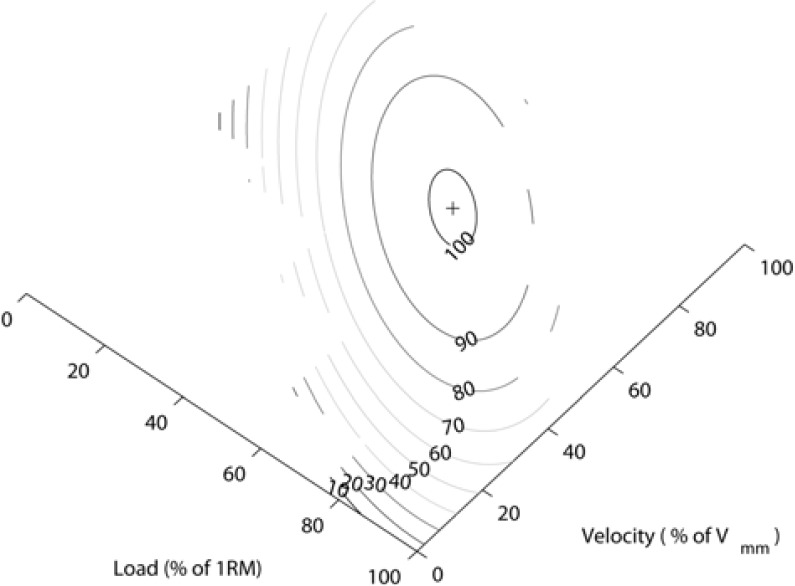
The contour lines of the linear regression model for the complete positive power movement.

Figure 7 presents the developed linear regression model, while Figure 8 presents the contour lines of this model. The optimal load for this model is 50.34% of 1RM, while the optimal velocity is 70.13% of *v*_mm_.

## Discussion

The first aim of this study was to create function which describes the relationship between relative load, velocity and power output for bench press exercise of elite soccer players. We developed a regression model to describe the relationship between maximum dynamics strength, maximum power output, maximum velocity, load, velocity and power. The model was consistent with the observed data. The coefficient of determination between the observed data and model was 0.74 for the acceleration phase of the movement and 0.75 for the complete positive power movement thus suggesting that the regression model corresponds very well with the observed data. The model indicates the dependence of the power output on external load and includes information about the speed of movement for the given exercise. It can be used not only for setting the optimal load at which maximal power output is reached, but also for the calculation of any parameter of load, power, velocity, maximum power, maximum velocity or one repetition maximum. This model describes the relationships of power output, velocity and load in a complex specific bench press exercise unlike Hill’s (1938) model which describes the relationships of power, velocity and load for stimulated frog muscle contractions. Jandacka and Vaverka (2008) created a model of the dependence of the power on the load for bench press but this model does not include information on the velocity of the movement.

The second objective of this study was to determine the optimal combination of relative load and velocity which predisposes maximal mechanical power in highly trained soccer players. The optimization approach tries to answer the following question: Which set of models will produce a result that will maximize mechanical power output during the bench press exercise (Caldwell 2004)? Hill’s (1938) equation implies that maximum mechanical muscle power output is reached approximately by one third of the maximum strength and maximum immediate velocity. But for the acceleration phase of the bench press lift our model predicts that the optimal load would be 40.43% (lower confidence limit 39.79% and upper confidence limit 42.24%) of 1RM, while the optimal mean velocity would be 74.58% (lower confidence limit 70.32% and upper confidence limit 87.14%) of *v*_mm_. The optimal mean velocity of movement determined in our study is significantly factually different to the optimal 30% of the maximum velocity peak determined by Hill (1938). To estimate the optimal load and velocity to achieve maximum muscle power during the complete positive power of the bench press lift our model predicts that the optimal load would be 50.34% (lower confidence limit 48.37% and upper confidence limit 60.44%) of 1RM, while the optimal velocity would be 70.13% (lower confidence limit 65.62% and upper confidence limit 98.15%) of *v*_mm_. With regard to the higher mean muscle activity during the acceleration phase of the movement (Newton et al. 1997), we should logically use loads that are within the range determined on the basis of the mean power output calculated from the acceleration phase of the movement to develop power output by dynamic effort strength training (Jandacka et al. in press). On the contrary, the optimal load determined in the range where the positive instantaneous power output is demonstrated should be used for training focused on the performance of the maximal amount of mechanical work in the shortest period possible during one lift (Jandacka et al. in press). In addition, according to Jidovtseff et al. (2009) the relationship between power, velocity and load could be used for setting optimal intensities during strength training.

The third objective of the study is to propose exercise intensity zones for resistance training of soccer players on the basis of the determined regression function. Miller (1997) determined four general zones of strength training on the basis of the knowledge of one repetition maximum: the zone of maximum velocity from 0 to 30% of 1RM, the zone of power -velocity from 30 to 50% of 1RM, the zone of power -strength from 50% of 1RM to 80% of 1RM and the zone of maximum strength from 90% of 1RM to 100% of 1RM. The loads determined by Miller (1997) in the zone of maximum velocity would correspond to the velocity of 80 to 100% of maximum mean velocity and power outputs from 64 to 97% of the maximum power output according to our model. Thus, this zone of loads corresponds with the requirements of the speed training from the point of the movement velocity and partially also to the power output training as 30% 1RM already reaches 97% of the power output maximum. According to Jidovtseff et al. (2009), maximum power should also be developed in the area power from 80 to 100%. The field of velocity-power corresponds with the relative load from 10 to 40% 1RM and velocity from 75 to 97% of *v*_mm_ according to our model. The boundary between the velocity-power zone and strength-power zone is the extreme of the function of the power output relationship to the applied load and in our case also to velocity according to Jidoffcev et al. (2009). The zone of strength-power corresponds with the relative load from 40 to 70% maximum and velocity from 38 to 75% maximum. According to our model of the acceleration phase, the speed from 11 to 32% of maximum mean velocity and power from 25 to 66% of maximum power output would correspond with the loads in the zone of maximum strength determined by Miller (1997). The determined regression function thus helped us understand that the boundary between the individual training zones determined only on the basis of the knowledge of maximum load expressed by one repetition maximum^21^, or power -velocity or power -load relationships (Jidoffcev et al. 2009), cannot be univocally set so that the intensity of the training only corresponds with the development of maximum velocity, maximum power or maximum load. The power -velocity-load relationship in the bench press exercise is probably close to the quadratic relationship as seen in Figure 4 and 7.

### Limitations

Our approach neglected the power associated with the motion of individual segments relative to the center of gravity of the body (rotational movement of body segments), power of antagonistic muscles, specific action of two joint muscles or elastic energy (Zatsiorsky 2002). The main topic of interest in this study is not the total power done on the body-barbell system but the mechanical power expended by the sources. This simplification could affect the determination of the optimal load for the maximal power output and consequently strength training zones. It should be pointed out that restrictions were induced by a chosen group of subjects who participated in the study and the analysis of the vertical movement of the center of gravity during free weight form only.

### Practical applications

The power is clearly defined by velocity and force. Thus, it is more sensible to use the three-dimensional power -velocity-load relationship rather than two-dimensional power -load and velocity-load relationships for the individual setting of training zones recommended by Jidoffcev et al. (2009). That is why we developed a regression model to describe the relationship between relative strength, relative power and relative velocity. This dependence seems to be quadratic, which is confirmed by the consistence of the model with the measured data. The model allowed us to set the optimal load for the dynamics effort strength training during a bench press exercise. The optimal load for reaching maximum power output suitable for the dynamics effort strength training for trained soccer players with a similar strength status as the subjects of the study would be 40% of 1RM, while the optimal mean velocity would be 75% of *v*_mm_. The optimal load determined from the entire extent of the positive power output suitable for training focused on the performance of the maximal amount of mechanical work in the shortest period possible during one lift would be 50% of 1RM, while the optimal velocity would be 70% of *v*_mm_. According to our model of the acceleration phase, the velocity from 80 to 100% of the maximum mean velocity and power outputs from 64 to 97% of the maximum power output would correspond with the load from 0 to 30% 1RM in the maximum velocity training zone. The velocity-power zone according to our model corresponds with the relative load from 10 to 40% 1RM and velocity from 75 to 97% of *v*_mm_. The strength-power zone corresponds with the relative load from 40 to 70% 1RM and velocity from 38 to 75% of *v*_mm_. The velocity from 11 to 32% of the maximum mean velocity and power outputs from 25 to 66% of the maximum power output would correspond with the loads above 80% 1RM in the maximum strength zone according to our model of the acceleration phase. Such precisely defined zones of strength training can help the trainer to rationalize the setting of the intensity of training as well as to control the effort of the particular athlete during the strength training.

## Conclusion

- The relationship between the relative power, relative load and relative velocity of the lift during the bench press exercise may be described by the quadratic function.
- This quadratic function may be used for the determination of the optimal load and velocity in order to reach the maximum mechanical power output.
- This quadratic function may be used for the determination of the exercise intensity zone for specifically focused strength training.
